# Post-Acute COVID-19 Syndrome (PACS) and Exercise Interventions: A Systematic Review of Randomized Controlled Trials

**DOI:** 10.3390/sports13090329

**Published:** 2025-09-15

**Authors:** Valentina Presta, Alessandro Guarnieri, Fabiana Laurenti, Salvatore Mazzei, Orsola di Martino, Marco Vitale, Giancarlo Condello

**Affiliations:** 1 Department of Medicine and Surgery, University of Parma, 43126 Parma, Italy; valentina.presta@unipr.it (V.P.); alessandro.guarnieri@unipr.it (A.G.); fabiana.laurenti@unipr.it (F.L.); salvatore.mazzei@unipr.it (S.M.); orsola.dimartino@unipr.it (O.d.M.); 2 Department of Neuroscience, Biomedicine and Movement, University of Verona, 37134 Verona, Italy; 3 Faculty of Medicine and Surgery, University Vita-Salute San Raffaele, 20132 Milan, Italy; vitale.marco@hsr.it

**Keywords:** long-COVID, exercise protocol, quality of life, physical fitness, training

## Abstract

The aim of this systematic review (PROSPERO registration number CRD42024517069) was to investigate the effectiveness of exercise interventions in Post-Acute COVID-19 Syndrome (PACS). We searched on several databases and followed the PRISMA guidelines (Preferred Reporting Items for Systematic Reviews and Meta-Analyses). We included randomized controlled trials that evaluate exercise interventions in adults (40–60 years old) diagnosed with PACS. The outcomes of interest were health-related quality of life (HRQoL) and functional fitness. Twenty studies were included after screening. Thirteen and fourteen studies were rated as “low” risk for HRQoL and functional fitness outcomes, respectively. Based on the evidence, an 8-week exercise protocol of aerobic training in combination with strength-based and breathing exercises was found to be safe and feasible while improving quality of life and functional fitness in people with PACS. Telerehabilitation can also be an option to avoid contagion and physical contact with the same beneficial effects. Future research should expand the knowledge about other types of exercise (i.e., water-based exercises) with high-quality trials and consider whether findings could be potentially transferable to recovery from a wider spectrum of viral infections.

## 1. Introduction

After the onset of the severe acute respiratory syndrome coronavirus 2 (SARS-CoV-2) infection, long-term consequences emerged as latent symptoms for people recovered from the initial infection. Post-Acute COVID-19 Syndrome (PACS) is characterized by persistent, relapsing, or new symptoms that occur 30 or more days after the acute phase of SARS-CoV-2 infection, with a broad spectrum of physical and mental health manifestations that reduce quality of life [1]. PACS was first described in a Delphi study conducted by the World Health Organization (WHO) and then included in the International Classification of Diseases 10 and 11 (ICD-10 and ICD-11). People experiencing symptoms 4 weeks after the onset of the SARS-CoV-2 infection are thought to be affected by COVID-19 late sequelae or long COVID [2].

Long COVID is estimated to affect at least 10% of those recovering, corresponding to about 65 million people worldwide, with more than 200 reported symptoms including fatigue, cognitive deficits, sleep disorders, muscle weakness, and psychological problems [3,4,5]. A recent meta-analysis confirmed persistent symptoms for a minimum of 28 days post-infection, with COVID-19 survivors experiencing unresolved symptoms around four months after infection [6]. Possible mechanisms involve viral persistence, immune dysregulation, and blood cell changes, resembling other post-viral syndromes [7]. A total of 9.8% of patients with mild COVID-19 reported persistent symptoms one year post-infection, with an early phase (30–180 days) marked by hair loss, chest pain, cough, myalgia, and respiratory disorders, and a late phase (180–360 days), where most symptoms resolved [8]. Symptom severity defines three phenotypes (mild, moderate, severe), while clinical clusters (fatigue-like, respiratory, chronic pain, neurosensorial) describe different trajectories, with overlap indicating greater severity and reduced quality of life [9,10]. Stratifying patients by these profiles could help design targeted interventions and rehabilitation [9].

Several risk factors related to PACS were identified, including age (≥40 years old), female sex, frailty, emergency visits, and hospitalization due to COVID-19. Conversely, vaccination appears to reduce long COVID incidence, with a lower risk of dyspnoea [8,11], and increased protection with more doses. Identifying at-risk populations can contribute to the understanding and prevention of long COVID [11], addressing also the reduced quality of life [12].

The heterogeneity of symptoms that occurs in long COVID complicates the diagnosis and management of the condition. Exercise has emerged as a promising intervention [13]. However, evidence indicated some barriers to engaging in exercise and a lack of guidance regarding physical activity, pointing to the necessity for tailored exercise programs in individuals with long COVID [14]. Wright et al. [15] reported a marked decline in physical activity levels among individuals with long COVID and symptom exacerbation in response to physical exertion, highlighting the development of safe and effective exercise interventions [15]. Physically active individuals with a confirmed diagnosis of COVID-19 had a significantly lower risk of hospitalization, fewer hospital days, less respiratory distress, and a decreased need for oxygen support compared to sedentary people post-infection [16]. Similarly, physically active people with long COVID experienced lower hospitalization rates due to symptom exacerbation and a related better management, preventing disabilities and reducing the need for further interventions or medications [17].

Some trials have investigated the effects of physical exercise programs of variable duration and type in individuals with PACS, showing improvements in symptoms and quality of life. Some interventions, lasting from 1 to 2 weeks, assessed moderate-intensity aerobic exercise or a combination of endurance and balance training, yielding improvements in cardiorespiratory fitness and functional status [18], symptoms severity and immune function [19]. An 8-week protocol of aerobic training combined with strengthening exercises improved cardiorespiratory fitness, but neither quality of life nor persistent symptoms (dyspnoea, fatigue) improved [20]. Conversely, strengthening exercises of respiratory muscles improved quality of life but not exercise tolerance or lung function [21]. Finally, the current limitations in robust evidence make it difficult to publish effective interventions for long COVID [22]. Therefore, this systematic review investigated the impact of exercise in long COVID management, with a specific focus on the type of intervention and its effects on physical function and health-related quality of life.

## 2. Materials and Methods

The protocol of the systematic review was previously registered on PROSPERO (International Prospective Register of Systematic Reviews, CRD42024517069). The review followed the Preferred Reporting Items for Systematic Reviews and Meta-Analysis (PRISMA) [23]. The review question was “Is there scientific evidence concerning the effectiveness of exercise interventions in Post-Acute COVID-19 Syndrome”? The patients, interventions, control, outcome, and study design (PICOS) format [24,25] was based on the following:

Population

This systematic review included studies focused on adults aged between 40 and 60 years old, with a confirmed diagnosis of PACS regardless of whether they required hospitalization for acute COVID-19 infection. Evidence showed that individuals aged 36–50 years had the highest proportion of long COVID diagnoses [7]. Similarly, a study characterizing PACS reported that adults aged 40–60 years were the most affected [10]. The studies including children, adolescents, and the elderly (over 60 years old), and people with ongoing physical or mental illness were excluded. Moreover, due to the heterogeneity in the definition of PACS, we did not apply restrictions related to diagnostic criteria or to the duration of symptom persistence, in order to include a larger number of studies potentially relevant to exercise interventions.

Intervention(s)

This review considered studies without specific restrictions regarding exercise interventions to include all studies that addressed any type of physical exercise. Studies that were not focused on exercise intervention were excluded.

Comparator(s)

This review considered studies that compared exercise interventions to any other standard management or care of PACS-diagnosed people.

Outcomes

Health-related quality of life (HRQoL) and functional fitness were identified as outcomes. Therefore, the studies including exercise interventions to determine their impact on the quality of life and/or functional fitness of individuals with PACS were included in the systematic review.

Studydesign

This systematic review included only randomized controlled trials (RCTs).

### 2.1. Search Strategy and Data Extraction

The literature search was performed through the indexed databases (BASE, EBSCO, EMBASE, PubMed, ScienceDirect, Scopus, and Web of Science) and clinical registers (Clinical Trials and Cochrane Library Register). The relevant articles published up to July 2025 were searched following a combination of keywords (e.g., Post-Acute COVID 19 Syndrome, long COVID, exercise, physical activity, training, health related quality life, physical fitness) and according to the specific search strategy of databases (e.g., possibility to use or not the Boolean operators and/or truncation of search terms). Once duplicates were removed from the total of RCTs identified, the screening process was conducted independently by two authors, and disagreements were verified by a third author. After the screening of title and abstract and availability check of the English full text, the remaining RCTs were considered eligible and further evaluated following the stated inclusion criteria. The references of the selected article were also considered. The relevant information from the selected articles was extracted and recorded in a spreadsheet (Excel^®^ file, Microsoft^®^ Excel^®^ version 2502 Build 16.0.18526.20546, 64 bit), including study design and population demographics, details of the exercise interventions and comparators, outcome measures, and evaluation of the risk of bias.

### 2.2. Risk of Bias Assessment

A revised Cochrane risk of bias (RoB 2, version of 22 August 2019) tool for randomized trials was used to evaluate the quality of the included studies [26]. This tool considered the risk of bias arising from five domains: randomization, deviations from intended interventions, missing or incomplete outcome data, outcomes measurement, and selection of reported results. Each study was analysed for each outcome and domain with an algorithm-based approach, guided by responses to signalling questions. The quality assessment was performed by two authors independently, and disagreements were verified by a third author. An overall judgement was determined for each outcome and study as having “low”, “some concern”, or “high” risk of bias.

## 3. Results

**Figure 1:**
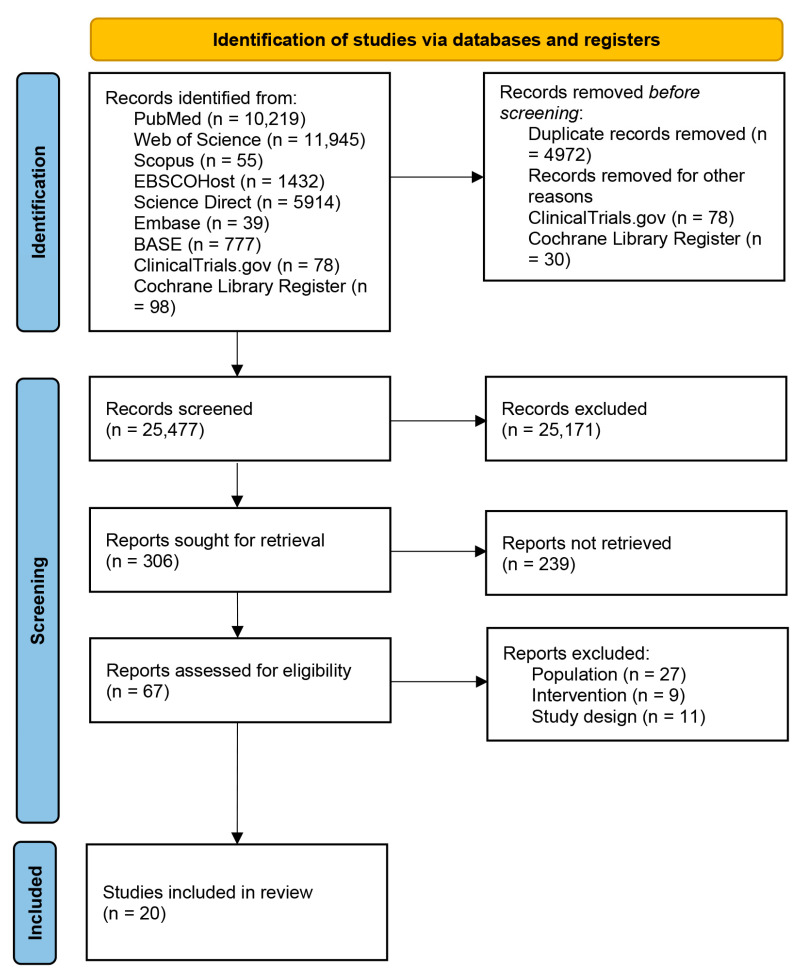
Preferred Reporting Items for Systematic Reviews and Meta-Analysis (PRISMA) flow diagram.

A total of 30,557 articles were identified from databases and registers (Figure 1). Before screening, 4972 duplicates were excluded. All the trials identified from ClinicalTrials.gov were excluded due to incomplete or unpublished results. Non-interventional studies, non-randomized trials, or out-of-scope studies were also excluded. Thirty studies were also excluded from the Cochrane Library Register due to unpublished results. Finally, a total of 25,171 articles were excluded for multiple reasons according to the PICOS framework (i.e., non-long-COVID population, non-interventional studies, non-controlled trials, studies that did not investigate the outcomes of interest, study designs other than RCTs). The remaining 306 articles were screened for title and abstract, and 67 were assessed for eligibility. Among these, 27 articles were excluded because the investigated population was identified as post-discharge patients, survivors, or patients recovered from COVID-19, thus without a diagnosis of PACS; 9 studies did not consider exercise intervention (e.g., electrical stimulation, vocal breath, etc.); and 11 studies were excluded due to the study designs (e.g., protocols, congress abstract, etc.). The remaining 20 articles were included in this systematic review.

### 3.1. Studies Characteristics

One study was published in 2022 [27], seven studies were published in 2023 [28,29,30,31,32,33,34], nine studies were published in 2024 [35,36,37,38,39,40,41,42,43], and three studies were published in 2025 [44,45,46].

The sample size of the included studies ranged from 14 participants [42] to 585 participants [41]. Females were equally or mainly represented in all the studies except for [30,32,34,40] and Ref. [45], which included females at a lower percentage as compared to males.

The diagnosis of PACS was generally consistent across studies, relying on the persistence of symptoms lasting 3 months or more. Three studies recruited individuals with symptoms of PACS lasting from 4 to 6 weeks after SARS-CoV-2 infection [28,31,40]. One study did not report the duration of symptoms in the investigated population [34]. One study diagnosed PACS according to the dyspnea modified medical research council (mMRC) scale [43].

### 3.2. Exercise Interventions

The duration of exercise interventions ranged from a minimum of 2 weeks [31] to a maximum of 12 weeks [39,40,46], with a mode of 8 weeks in half of the studies. Where specified, the frequency of training sessions was three times per week with a duration of 60 min. In half of the studies, the execution of the exercise protocol was supervised via an app (Fisiotrack—https://fisiotrack.com/ -or other mobile phone application not specified) [28,40] or conducted in groups or in one-to-one online videoconferences. However, types of exercise differed across studies. Four studies [27,29,35,46] investigated the effects of concurrent training (i.e., resistance training in combination with aerobic exercise), setting the intensity of resistance training at 50% of one repetition maximum (1RM), at a moderate level for the aerobic training, and, once a week, light intensity was additionally planned. Similarly, a combination of aerobic exercise and resistance training was planned in seven studies [32,34,36,38,39,42,45]. Intensity of exercise was set at a moderate level, expressed by 60–70% of VO_2_max [32,36] or of the maximum heart rate [34,39], or by the Borg or OMNI Scale ranging from 4 to 6 and 8, respectively [35,42]. Breathing exercises were included in four studies in combination with aerobic training [37], strength-based exercises [31,45], or with pilates and yoga at variable intensities [41]. The other studies investigated the effects of (i) functional exercise (i.e., low-intensity strengthening exercise for large muscle groups) [28]; (ii) continuous aerobic training at 50% of workload [30]; and (iii) virtual reality-based program including high-intensity cycloergometer training [33].

The control groups varied across studies. Three studies compared the exercise group to general guidance on physical exercise and healthy habits [36] or WHO guidelines “Support for rehabilitation: Self-management after COVID-19 related illness” [27,29]. The other studies compared the experimental group to (i) no intervention, wait list, or usual care [31,35,37,38,39,40,41,44,45,46], (ii) exercise in a different setting (i.e., at hospital) [34], (iii) different exercise delivery (i.e., unsupervised, or traditional methods) [32,33,42,43], or (iv) different exercise protocols (i.e., aerobic or interval training) [28,30].

### 3.3. Outcomes Measured

The outcomes related to HRQoL and functional fitness were both investigated in 16 of 20 studies. Four studies investigated only the functional fitness as an outcome [31,33,38,43]. One study investigated the HRQoL as an outcome only [32]. The HRQoL was mainly evaluated with the European Quality of Life 5 Dimensions 5 Levels (EQ-5D-5L). The other studies used the 36-item short form health survey (SF-36) [30,39,44,46] or the shorter version (SF-12) [27,29,32,35]. Three studies used the Quality of Life Questionnaire (VQ11) [34], the St George’s Respiratory Questionnaire [37], and the World Health Organization Quality of Life Bref (WHO-QoL-BREF) [40], respectively. The functional fitness was evaluated with the Cardiopulmonary Exercise Testing (CPET) in eight studies [27,29,30,38,39,40,44,46], and in seven studies, the functional fitness was evaluated with the sit-to-stand test [28,34,42,43,44] or hand grip test [35,45]. Three studies used the 6 min walking test (6MWT) [31,33,37], and two studies used the International Physical Activity Questionnaire (IPAQ) [37,40].

All the included studies in this review were RCTs. Only one study was double-blind [31], and four were multicenter studies [32,35,37,41] (Table 1 and Table 2).

### 3.4. Risk of Bias

**Figure 2:**
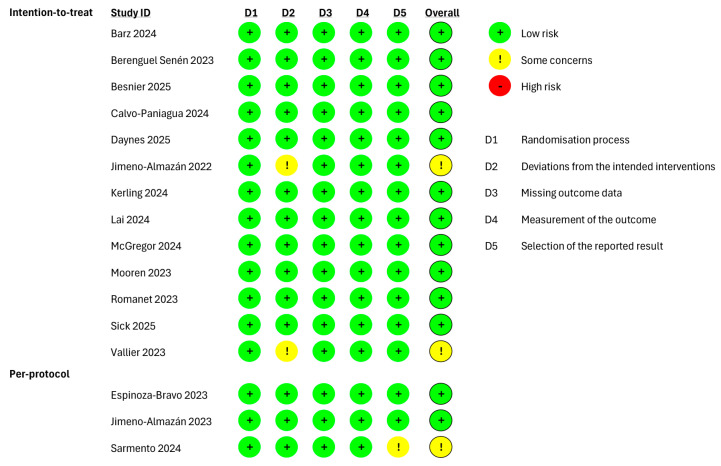
Risk of bias of the included studies evaluating the health-related quality of life (HRQoL) as outcome and performing an “intention-to-treat” and “per-protocol” analyses. Barz 2024 [35], Berenguel Senén 2024 [36], Besnier 2025 [44], Calvo-Paniagua 2024 [37], Daynes 2025 [45], Jimeno-Almazán 2022 [27], Kerling 2024 [39], Lai 2024 [40], McGregor 2024 [41], Mooren 2023 [30], Romanet 2023 [32], Sick 2025 [46], Vallier 2023 [34], Espinoza-Bravo 2023 [28], Jimeno-Almazán 2023 [29], Sarmento 2024 [42].

**Figure 3:**
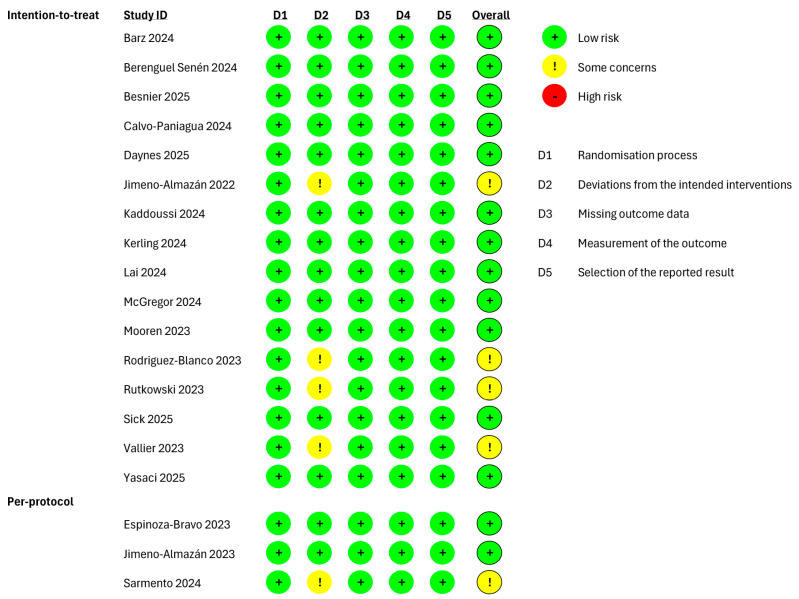
Risk of bias of the included studies evaluating the functional fitness as outcome and performing an “intention-to-treat” and “per-protocol” analyses. Barz 2024 [35], Berenguel Senén 2024 [36], Besnier 2025 [44], Calvo-Paniagua 2024 [37], Daynes 2025 [45], Jimeno-Almazán 2022 [27], Kaddoussi 2024 [38], Kerling 2024 [39], Lai 2024 [40], McGregor 2024 [41], Mooren 2023 [30], Rodriguez-Blanco 2023 [31], Rutkowski 2023 [33], Sick 2025 [46], Vallier 2023 [34], Yasaci 2025 [43], Espinoza-Bravo 2023 [28], Jimeno-Almazán 2023 [29], Sarmento 2024 [42].

The risk of bias of the included studies was assessed (Figure 2 and Figure 3). It is important to mention that 16 out of 20 studies evaluated both outcomes; thus, the assessment of the bias risk was performed separately. For studies evaluating only one between HRQoL and functional fitness, the assessment of bias risk was performed for the evaluated outcome only. Moreover, regardless of the investigated outcome, 3 out of 20 studies aimed at evaluating adherence to the intervention (“per-protocol effect”) [28,29,42], whilst the remaining studies aimed at evaluating the assignment to intervention (i.e., the “intention-to-treat” effect). Therefore, the risk of bias was independently assessed according to the nature of the effect of interest.

Regarding the studies investigating the HRQoL with an “intention-to-treat” analysis, the risk of bias was judged as “low”, except for two studies in which “some concerns” were identified due to deviations from the intended interventions [27,34]. Among the studies performing a “per-protocol” analysis, a bias in the selection of the reported result was observed in only one study [42].

The studies investigating the functional fitness, with an “intention-to-treat” analysis, were rated as “low”. Four studies were rated with “some concerns” [27,31,33,34], and Ref. [42] was rated with “some concerns” because of deviations from the intended interventions among the studies performing a “per-protocol” analysis.

The low-to-moderate risk of bias across studies supports the reliable effects of exercise interventions on HRQoL and functional fitness. However, the presence of some concerns may have influenced the observed effects. Consequently, while the results appear consistent, the confidence in the effect estimates remains moderate.

## 4. Discussion

The aim of this systematic review was to review the scientific literature to analyse whether any type of exercise intervention could improve health-related quality of life and physical function in people with a PACS diagnosis. A total of 20 RCTs was included in the systematic review, published from 2022 to 2025. The years of publication are consistent with the first characterization of PACS or “long COVID”. In the second part of 2020, a post-COVID syndrome was only suspected [47], and then it was preliminarily confirmed with a case series study [48] in which fatigue and dyspnoea were identified as persisting symptoms in the follow-up (i.e., after the discharge of acute COVID-19 illness). The RCTs included in this review recruited participants with a confirmed diagnosis of PACS according to [2]—3 months after SARS-CoV-19 infection. However, two studies referred to PACS involving individuals with persisting symptoms for at least 40 days [31] or 6 weeks [28], and in one study, the duration of symptom persistence was not specified [34]. The heterogeneity in the PACS definition can be explained by the fact that the authors have based their work on earlier studies, which described the condition in different ways [48,49], and a lack of worldwide consensus at the time of study publication. These initial differences in definitions have led to a variety of methods and understandings of how long COVID has been studied and managed. Nevertheless, as knowledge increased, the clinical definition of PACS evolved, and it is still ongoing [50].

The frequency, setting, and delivery of exercise protocols were not comparable to each other, even though the type of exercise protocol was similar among some studies. Notably, in half of the studies, the exercise delivery was totally remote or in combination with a face-to-face program (i.e., telerehabilitation) [28,31,34,37,39,40,41,42,43,45]. This aligns with the guidelines of the time, which recommended avoiding physical contact to prevent contagion. Studies have demonstrated that telerehabilitation can improve quality of life and functional capacity in patients with COVID-19 sequelae, with favourable outcomes in terms of costs and health benefits. These findings suggest that telerehabilitation could be a valuable tool for providing equitable rehabilitation options to a broader patient population [51]. In the remaining studies, where the protocol was administered in person (at the hospital), aerobic activity was the most frequently investigated, but in different forms (e.g., virtual reality, aerobic versus usual care group, interval versus continuous training) [30,32,33]. Ref. [36] evaluated a combination of inpatient and home-based protocol in which the aerobic training was performed at home. Separately, two studies evaluated concurrent training [27,29], but in one protocol, it was also combined with inspiratory muscle training [29]. Regardless of whether the exercise protocol was conducted in person or remotely, all the studies reported positive results or feasibility (i.e., higher percentage of adherence). Aerobic training could be performed as moderate interval or continuous training, showing similar positive results. However, functional training showed slightly better results when compared with aerobic training or as part of concurrent training, whilst virtual reality did not yield greater results than traditional methods. However, findings related to aerobic training, even in combination with other types of training, are consistent with what is reported in the literature [52,53]. Some studies were excluded from this review due to study design (i.e., non-RCTs, protocols, reviews). Moreover, this review did not include water-based exercise, for which some existing literature has reported benefits in managing PACS symptoms and reducing hospitalization over the long term [17,54]. Finally, two studies focused on breathing exercises and were aimed at improving the respiratory function in PACS individuals. Specifically, a 2-week protocol of breathing- and strength-based exercises was clinically effective [31] as well as an 8-week protocol consisting of breathing exercises, pilates, and yoga disciplines at variable intensities [41]. These findings were also previously reported by [55], although their results were not specific to the PACS condition, and the effectiveness of yoga alone remains to be demonstrated. Therefore, a multi-training approach could be effective in improving the recovery of people with PACS [56]. This could be explained by the release of exerkines—namely signalling moieties released during exercise—with a crosstalk effect between multiple body systems, and it serves as a strategy in the treatment of long COVID [57]. Furthermore, it has been demonstrated that physically active people with PACS recover quickly and fully as compared to sedentary individuals with PACS, confirming that exercise can mitigate the debilitating symptoms of PACS [58]. The diversity of control conditions across studies may have affected the observed results. Comparisons of no-intervention or waitlist controls with alternative exercise modalities or educational guidance may overestimate or underestimate the reported benefits. Variations in execution modalities within control groups could limit direct comparisons and the generalizability of results.

The quality of life was one of the outcomes investigated in this review because PACS-related symptomatology has been demonstrated to reduce the wellbeing and lifestyle in people experiencing this condition [9,10]. In fact, most of the included studies evaluated whether an exercise protocol could influence HRQoL, and almost all reported related improvements in people with PACS. Ref. [32] did not detect significant changes in quality of life when comparing the same aerobic training but supervised by a general or specialized physiotherapist. However, it should be noted that the assessment of HRQoL was performed with the SF-12, consisting of a mental and physical component, and significant changes were, nevertheless, reported for the physical part of the survey. In this regard, the other studies that used the SF-12 [27,29,35] reported positive results in both the physical and mental components of the survey. However, the exercise interventions were shorter, and the studies were not multicentric; thus, the results were not applicable to a larger and more diverse sample. Studies that used the longer version of the same questionnaire (SF-36) [30,39], with similar sample size and duration of the exercise protocol [39,46], reported significant improvements in HRQoL. The SF-36 appears more sensitive in capturing changes in both physical and mental health domains, while the shorter version may be less responsive to subtle improvements, especially in physical function or fatigue. Compared with the other tools used (EQ-5D-5L and PROMIS), current evidence is still limited on their responsiveness in PACS. Overall, the SF-36 may be preferable for detailed evaluation, whereas the SF-12 can be considered for practical assessment [59,60,61]. Therefore, given that the quality-of-life assessment methods varied across the included studies, the research methodology may have influenced the outcome of these studies. Similarly, the functional fitness assessment varied across studies. Notwithstanding, the outcome-related parameters were improved in all the included studies regardless of the exercise protocol characteristics (excluding virtual reality, which showed no superior change compared to traditional methods) [33]. The overall low risk of bias identified across studies indicates that the quality of the studies is mostly high. However, in the studies where “some concerns” were identified, the reason was primarily attributable to masking procedures, which were not always possible due to the nature of the interventions [27,31,33,34,42].

Among the limitations of this review, the heterogeneity of the intervention protocols should be acknowledged. Most studies were not comparable in terms of frequency, duration, and modality. Therefore, despite overall positive trends, the heterogeneity of protocols prevented quantification of the magnitude of improvements across studies, making it difficult to establish which type of exercise should be considered superior. Moreover, the studies were conducted based on the knowledge available at the time, leading to differences in investigation methodologies and target population (e.g., diagnosis of PACS differed in terms of symptoms duration and clinical definition). Another limitation concerns the variability of outcome assessment tools. Quality of life was measured using different questionnaires (e.g., SF-12 vs. SF-36). Similarly, functional fitness assessments varied across studies. Moreover, these outcomes were not systematically analysed as a primary outcome, which may have underestimated their role in relation to exercise interventions. Finally, although overall dropout rates were below 37%, lack of adherence in some studies may still have affected outcome reliability and should be considered in the design of future studies.

Future research should address methodological biases and include preventive measures as exercise protocols to monitor long-term impacts and to improve understanding and treatment of PACS condition [7]. It is crucial to expand ongoing intervention studies based on current knowledge and generate high-quality evidence for diverse long-COVID populations [4,10]. Moreover, a cohort study [62] compared long-term health outcomes following COVID-19 illness and seasonal influenza, suggesting that the management of long COVID with exercise protocol could be transferable to seasonal influenza, hypothesizing the same improvement in functional fitness and HRQoL parameters. Further research could, therefore, be conducted, focusing on the influence of exercise interventions while recovering from viral infections.

## 5. Conclusions

The management of PACS still requires further in-depth studies regarding the treatment of symptoms according to its clinical definition. This review shows that an intervention protocol based on physical exercise is safe and feasible, also via telerehabilitation. The duration of the intervention can range from 2 to 12 weeks, although a higher adherence has been observed with 8-week protocols, three to five sessions per week, 1 h/session. An aerobic activity may provide benefits, as moderate or continuous, with greater effects if combined with strength-based exercises and/or as concurrent training. Further benefits can be achieved with specific respiratory muscle training (i.e., breathing exercises). However, not all exercise types consistently improved HRQoL or symptoms, highlighting the need for individualized protocols. Recommendations should consider clinical phenotype, baseline functional capacity, and adherence potential. Therefore, while exercise interventions appear promising, their prescription should be tailored and carefully monitored rather than universally recommended.

## Figures and Tables

**Table 1:** Study characteristics.

Reference	Aim	Population	Intervention	Comparison	Outcome	Study Design	Test	Results
Barz et al., 2024 [35]	To analyse the effect of an exercise program on fatigue and quality of life of people with PACS	118 participants, 53.5 ± 11.9 yr, 68.6% F	8 weeks of individualized concurrent resistance and aerobic training	Wait-list control group	Primary: change in FSS Secondary: changes in HRQoL and physical performance	Multi-centre, randomized	FSS, DSQ-14, hand grip, objective fatigability, Chester Step Test, SF-12	↓ fatigue score ↑ SF-12 score ↑ total steps 32.2% dropout (lack of time, other health issues)
Berenguel Senén et al., 2024 [36]	To assess the effect of therapeutic physical exercise program (TPEP) in people with PACS and exercise intolerance (EI)	50 participants, 47 ± 7.1 yr, 73% F	8-week TPEP, in-person and remotely monitored home sessions Aerobic training with increasing intensity and volume, twice-daily respiratory muscle training, neuromuscular training, HIIT, and core muscle exercises	Regular physical activity recommendations	Primary: change in VO_2_peak Secondary: change in quality-of-life scores, maximal inspiratory pressure, neuromuscular capacity, body fat percentage, mitochondrial function parameters	Single centre, randomized, open label	CPET, maximal inspiratory pressure, body composition analysis, neuromuscular assessment of various muscle groups, PCFS, EQ-5D-5L, and PHQ-9	↑ functional capacity ↑ EQ-5D-5L score ↑ strength ↓ fat percentage ↑ metabolic flexibility and mitochondrial function 26% dropout (refuse to participate after randomization, pregnancy, other health problems)
Besnier et al., 2025 [44]	To investigate the effect of cardiorehabilitation program in individuals with long COVID	35 participants, 53.2 ± 11.71 yr, 68.6% F	8 weeks of individualized and supervised exercise program, 3 times per week, combining aerobic and resistance training with inspiratory exercises	Wait list	Primary: change in VO_2_peak Secondary: submaximal CPET key parameters, physical functioning, quality of life, and change in PACS symptom	Two-arm randomized	CPET, TUG, STS-5, 6MWT, PCFS, MRC, SF-36	↑ cardiorespiratory fitness ↑ ventilatory efficiency ↑ physical functioning ↓ PACS symptoms 12.5% dropout (lost to follow-up)
Calvo-Paniagua et al., 2024 [37]	To evaluate the effect of a telerehabilitation exercise program on physical functioning and quality of life in PACS	64 participants, 50.1 ± 9.2 yr, 62.5% F	7 weeks, 18 sessions, 40 min per session in alternate days. General information about sessions, respiratory and aerobic training	No intervention (wait and see)	Primary: change in perceived physical exertion Secondary: change in dyspnoea severity, quality of life, endurance capacity	Prospective, multicentre, randomized clinical trial, two parallel groups	Modified Borg Dyspnea Scale, mMRC, SGRQ, 6MWT	↓ perceived physical exertion at rest and after physical activity, ↑ oxygen saturation at rest and after walking, ↓ dyspnea severity, ↓ SGRQ score (better health status) ↑ distance at 6MWT 0% dropout, all randomized participants received the intervention
Daynes et al., 2025 [45]	To compare face-to-face, remote or no exercise intervention in people with PACS	181 participants, 59 ± 12 yr, 45% F	Face-to-face: 8 weeks, twice weekly, 90–120 min per session of aerobic and resistance training Remote: as face-to-face but remotely monitored	Usual care	Primary: change in the incremental shuttle walking test Secondary: change in quality of life	Single-blind, three-arm randomized	SPPB, hand grip, EQ-5D-5L, PHQ-9, GAD-7, FACIT-FS, DSQ-14, Brief Pain Inventory, MoCA, mMRC, SARC-F, General Practice Physical Activity Questionnaire, Nijmegen Questionnaire	↑ incremental shuttle walking distance ↑ EQ-5D-5L score No difference between face-to-face and remote monitoring of physical activity 18% dropout (refused to participate after randomization, lost to follow-up)
Espinoza-Bravo et al., 2023 [28]	To compare the effects of telerehabilitation functional (FE) vs. aerobic exercises (AEs) training in PACS	43 participants, 42.4 ± 6.5 yr, 34 F (79.1%)	FE: 8 weeks, 3 sessions per week (Fisiotrack mobile phone application). Low-intensity strengthening exercise protocol for large muscle groups (body-weight squat, side squat, hip thrust, chest press, and rowing 2/3 sets × 10 reps, front plank 30”)	AEs: low-intensity walking protocol with weekly load adjustments, 25 min at week 1 up to 45 min at week 8	Primary: change in fatigue perception Secondary: change in dyspnoea, functional performance, quality of life, adherence to treatment	Prospective, randomized, single blind	FAS, London Chest ADL Scale, 30SST, PSS, HADQ, and EQ-5D-5L. Only after treatment: Patient Global Impression of Change Scale, System Usability Scale, and adherence to treatment	↑ stress symptoms and quality of life FE was more effective in improving fatigue perception and functional performance 10% dropout (lost to follow-up, other health conditions)
Jimeno-Almazán et al., 2023 [29]	To determine the effectiveness of physical exercise program in people with PACS	80 participants, 45.3 ± 8 yr, 55 F	Concurrent training with (CTRM) or without inspiratory muscle training (CT). Resistance training followed by moderate intensity training, and one day light intensity continuous training	Advise to follow the WHO guidelines: “Support for Rehabilitation: Self-Management after COVID-19-Related Illness” as a home-based program	Primary: change in cardiorespiratory fitness, and muscle strength Secondary: change in symptoms severity	Four-arm randomized, parallel groups	CPET, bench press, half squat, hand grip, SF-12, GAD-7, PHQ-9, mMRC, FSS, CFS, PCFS	↑ cardiovascular fitness and muscle strength ↓ dyspnea and perceived fatigue 7.5% dropout (lost to follow-up, other health problems)
Jimeno-Almazán et al., 2022 [27]	To evaluate the effect of a supervised therapeutic exercise program in people with PACS	39 participants, 45.2 ± 9.5 yr, 29 F (74.4%)	8 weeks, 3 days-a-week of concurrent training: 2 days of resistance training (50% 1RM, 3 sets, 8 repetitions, 4 exercises-squat, bench press, deadlift, and bench pull-combined with moderate intensity variable training), and one day of light intensity continuous training	Informed to follow the WHO guidelines (non-supervised): “Support for Rehabilitation: Self-Management after COVID-19 Related Illness”	Primary: change in symptoms severity, change in physical fitness, and cardiopulmonary function	Randomized controlled trial	SF-12, GAD-7, PHQ-9, mMRC, CFQ-11, FSS, DSQ-14 short form, PCFS, CPET, handgrip test, WHO GPAQ, resting ECG	↑ SF-12 score, cardiovascular fitness, muscular strength ↓ fatigue, depression, symptoms severity 2.6% dropout (lost to follow-up)
Kaddoussi et al., 2024 [38]	To evaluate the impact of cardiopulmonary rehabilitation program on aerobic capacity in people with long COVID	30 participants, 52.5 ± 14 yr, 53.3% F	6 weeks, 18 sessions, 3 sessions per week, 60–90 min, aerobic and resistance training	Usual level of sedentary activities	Primary: change of 6MWD Secondary: change in dyspnoea and spirometry parameters	Single-blinded randomized	Borg Scale, mMRC, spirometry, 6MWT, HR	↑ 6MWD ↓ dyspnea, HR No change in spirometric parameters 16.7% dropout (lost to follow-up)
Kerling et al., 2024 [39]	To assess the impact of remotely monitored exercise intervention on PACS symptoms	62 participants, 46 ± 12 yr, 42 F	3-month, home-based intervention including 150 min of moderate physical activity per week, and strength training	Usual lifestyle and daily activities	Primary: change in VO_2_peak Secondary: change in FAS score, quality of life; evaluation of work ability, and spirometric parameters	Prospective, randomized, parallel groups, single blind (assessor blind)	Exercise capacity (bicycle with gas exchange), fatigue, markers of HrQoL (SF-36) and mental health	↓ FAS score No changes detected in exercise capacity, quality of life, and work ability 14% dropout (refused to participate, lost to follow-up)
Lai et al., 2024 [40]	To investigate the effectiveness of a 12-week telerehabilitation program in people with PACS	182 participants, 39.85 ± 12.55 yr, 38.5% F	12 weeks telerehabilitation program (with mobile app monitoring), thrice/week, 40 min, endurance training	Usual lifestyles	Primary: change in cardiorespiratory fitness Secondary: evaluation of sleep quality, quality of life, amount of PA	Randomized controlled trial, parallel group	CPET, IPAQ, Self-Efficacy Exercise Scale, PSQI, WHO-QOL-BREF	↑ total PA, and sleep quality No differences in cardiorespiratory fitness, and quality of life 33% dropout (lack of time, lost to follow-up, other health issues)
McGregor et al., 2024 [41]	To evaluate the effect of remote rehabilitation program on quality of life of people with PACS	585 participants, 56 ± 12 yr, 52% F	8-week home-based, supervised, group exercise (breathing, Pilates, and yoga, at variable intensity)	Usual care (30 min of online education sessions)	Primary: assessment of quality of life Secondary: PROMIS^®^ subscores (depression, fatigue, sleep, physical function, etc.)	Multicentre, randomized, parallel groups	PROMIS^®^	↑ PROMIS^®^ score 24.4% dropout (lost to follow-up, refused to participate)
Mooren et al., 2023 [30]	To compare continuous vs. interval aerobic training in people with PACS	110 participants, 49.3 ± 11.8 yr, 38% F	Continuous training on cycloergometer, 3–5 sessions per week, 18 min, 50% of maximal workload	Interval training on cycloergometer, load = 60%, relief = 30%	Primary: change in VO_2_peak Secondary: submaximal oxygen uptake, quality of life	Prospective, two-arm randomized, parallel groups, open label	MFI-20, SF-36, WHO-5, WAI, CPET, laboratory parameters	↑ SF-36 score, cardiovascular fitness ↓ fatigue, anxiety, and depression 20.9% dropout (scheduling problems)
Rodriguez-Blanco et al., 2023 [31]	To investigate the effect of telerehabilitation in people with PACS	48 participants, 40.7 ± 13.4 yr, 26 F	14-day telerehabilitation program including 10 breathing- and strength-based exercises, 12 reps/exercise on consecutive days for 30 min	Usual daily activities with no additional physical exertion	Primary: change in fatigue perception, cardiovascular fitness, and dyspnea levels	Two-arm randomized, parallel groups, double blind	VAFS, 6MWT, 30STST, Borg Scale	↑ 6MWD, 30STST score ↓ Borg Scale score, VAFS 7.7% dropout (lost to follow-up)
Romanet et al., 2023 [32]	To evaluate the effects of exercise training rehabilitation (ETR) in people with COVID-19-related acute respiratory distress syndrome (CARDS)	60 participants, 58 ± 12 yr, 23 F (38%)	2 × 60 min sessions of aerobic training per week for 10 weeks, in combination with strength training	2 × 30 min sessions of aerobic training per week for 10 weeks, in combination with strength training (standard physiotherapy)	Primary: measurement of dyspnoea Secondary: measurement of functional dyspnoea, assessment of quality of life	Multicentre, two-arm randomized, parallel groups, single blind (assessor blind)	mMRC, SF-12	↓ dyspnea in ETR group vs. standard physiotherapy group No difference in SF-12 score, except for physical component (↑ following ETR vs. standard physiotherapy group) 0% dropout, all randomized participants received the intervention
Rutkowski et al., 2023 [33]	To evaluate the effect of virtual-based pulmonary rehabilitation program in people with PACS	32 participants 57.8 ± 4.9 yr, 20 F	VR-led 3-week, five-times-week of incremental cycloergometer training	Traditional (without VR) 3-week, five-times-week incremental cycloergometer training	Primary: assessment of lung function, exercise performance and chang in stress levels	Randomized controlled trial	6MWT, spirometry, PSS	↑ 6MWD ↓ PSS score No changes in spirometric parameters. Equal results in VR-led and traditional training 0% dropout, all randomized participants received the intervention
Sarmento et al., 2024 [42]	To assess the feasibility of a virtual pulmonary rehabilitation in individuals with PACS	14 participants, 49 ± 9 yr, 12 F (86%)	8-week virtual pulmonary rehabilitation (PR) program: supervised 30 min of resistance and aerobic exercises, three times a week, intensity at 4–6 of Borg Scale	Same as experimental group but not supervised (self-directed (PRSD))	Primary: feasibility assessment Secondary: evaluation of lung function, change in dyspnea, perceived fatigue, exercise capacity, and quality of life	Two-arm randomized	Spirometry, Borg scale, FSS, DSQ short form, 1STS, EQ-5D-5L, COPM	Virtual PR is feasible and safe ↑ 1STS score No differences in the other investigated outcomes 26.3% dropout (lost to follow-up, other medical issues)
Sick et al., 2025 [46]	To investigate the effects of endurance vs. concurrent exercise in individuals with PACS	66 participants, 41.2 ± 12.3 yr, 78.6% F	12 weeks, 3 session per week, endurance exercise or concurrent exercise (i.e., combination of endurance and resistance exercise within the same session)	Non-exercise intervention group	Primary: change in VO_2_peak Secondary: evaluation of strength, heart rate variability, PACS symptoms, quality of life	Single centre, randomized, parallel groups	CPET, leg press, handgrip test, HR variability, FSS, mMRC, SF-36	Following both endurance and concurrent were reported: ↑ SF-36 score ↑ VO_2_peak ↓ PACS symptoms ↓ FSS score Following concurrent training only ↑ lower body strength and dyspnea 36.4% dropout (lost to follow-up)
Vallier et al., 2023 [34]	To compare home-based vs. inpatient rehabilitation in people with PACS	17 participants, 54.8 ± 16 yr, 5 F	4 weeks, home-based protocol (HPR) composed of 16 aerobic sessions, 12 strength sessions and 4 relaxation sessions	4 weeks, inpatient protocol (IPR) as experimental group	Primary: change in dyspnea and perceived fatigue, quality of life, exercise capacity, pulmonary function	Randomized controlled trial	mMRC, MFI, VQ11, 6MWT, 1STS test, squat jump, spirometry	↑ 6MWD, 1STS, and squat jump score ↑ VQ11 score No changes in spirometric parameters and dyspnea levels Equal improvements following HPR or IPR, except for fatigue reduction (IPR only) 0% dropout, all randomized participants received the intervention
Yasaci et al., 2024 [43]	To evaluate the effects of telerehabilitation program in people with PACS.	60 participants, 56 ± 11.4 yr, 51.6% F	6 weeks, 2 sessions per week, 45 min per session, including breathing, relaxation, range-of-motion, walking, and wall-squatting exercises	Unsupervised home training	Primary: evaluation of dyspnea level, pain severity, functional status, change in sleep quality, anxiety, and depression status	Single centre	mMRC, NPRS, 5-STS, PSQI, HADS	↓ dyspnea and pain intensity ↑ 5STS, PSQI and HADS score 6.3% dropout (lost to follow-up)

Footnotes: ↓, reduction; ↑; improvement; 1RM, one-repetition maximum; 1STS, one minute sit to stand test; 6MWT, 6 min walking test; 30SST or 30STST, 30 s standing test; ADL, activities of daily living; AEs, aerobic exercises; CARDS, COVID-19-related acute respiratory distress syndrome; CFS, Chalder Fatigue Scale; COPM, Canadian Occupational Performance Measure; CPET, cardiopulmonary exercise testing; CT, concurrent training or continuous training in [30]); CTRM, concurrent training with inspiratory muscle training; DSQ-14, DePaul Symptom Questionnaire; ECG, electrocardiogram; EI, exercise intolerance; EQ-5D-5L, European Quality of Life 5 Dimensions 5 Levels; ETR, exercise training rehabilitation; FAS, Fatigue Assessment Scale; FE, functional exercises; FSS, Fatigue Severity Scale; GAD-7, General Anxiety Disorder Questionnaire-7; GPAQ, Global Physical Activity Questionnaire; HADQ, Hospital Anxiety and Depression Questionnaire; HIIT, high-intensity interval training; HPR, home-based pulmonary rehabilitation; HR, heart rate; HRQoL, health-related quality of life; IPR, inpatient pulmonary rehabilitation; MFI-20, Multidimensional Fatigue Inventory; mMRC, modified Medical Research Council Dyspnoea Scale; PACS, post-acute COVID-19 condition; PCFS, post-COVID-19 functional status; PHQ-9, Patient Health Questionnaire-9; PR, pulmonary rehabilitation; PROMIS^®^, patient reported outcomes measurement information system; PRSD, pulmonary rehabilitation via self-directed; PSS, Perceived Stress Scale; SF-12, 12-item Short Form Health Survey; SF-36, Health Survey; TPEP, therapeutic physical exercise program; VAFS, Visual Analogue Fatigue Scale; VQ11, quality of life questionnaire; VR, virtual reality; WAI, Work Ability Index; WHO, World Health Organization; WHO-5, World Health Organization 5 wellbeing index.

**Table 2:** Comparative table summarizing modalities, dosage, and setting of exercise interventions.

Study	Intervention Type	Key Characteristics (Duration, Frequency, Intensity)
[28,30,39,40,45]	Aerobic training	Dur: 8–12 weeks Freq: 3–5×/week Int: Moderate (e.g., 50% max workload, Borg 4–6) or Interval (HIIT)
[28,31]	Resistance/Functional training	Dur: 14 days–8 weeks Freq: 3×/week to daily Int: Low (bodyweight, 2–3 × 10 reps)
[27,29,32,35,36,38,42,44,46]	Concurrent/Combined training	Dur: 6–10 weeks (mostly 8 weeks) Freq: 2–3×/week Int: Resistance: 50% 1RM or Borg 4–6; Aerobic: moderate (e.g., 60–70% VO_2_peak)
[28,31,37,40,43]	Telerehabilitation (mixed modalities)	Dur: 2–12 weeks Freq: Varies (daily to 3×/week) Int: Typically low-moderate; often includes breathing exercises, light strength, aerobic
[31,41,43]	Breathing exercises, mind–body	Dur: 8 weeks Freq: Not always specified Int: Variable, low
[33]	Virtual Reality	Dur: 3 weeks Freq: 5×/week Int: High (incremental cycloergometer)
[34]	Home-based vs. inpatient	Dur: 4 weeks Freq: Daily mix of sessions Int: Not specified

Footnotes: 1RM, one-repetition maximum; HIIT, high-intensity interval training.

## Data Availability

No new data were created or analyzed in this study. Data sharing is not applicable to this article.
